# Charged aerosol detector response modeling for fatty acids based on experimental settings and molecular features: a machine learning approach

**DOI:** 10.1186/s13321-021-00532-0

**Published:** 2021-07-15

**Authors:** Ruben Pawellek, Jovana Krmar, Adrian Leistner, Nevena Djajić, Biljana Otašević, Ana Protić, Ulrike Holzgrabe

**Affiliations:** 1 Institute for Pharmacy and Food Chemistry, University of Würzburg, Am Hubland, 97074 Würzburg, Germany; 2 Department of Drug Analysis, Faculty of Pharmacy, University of Belgrade, Vojvode Stepe 450, 11 221 Belgrade, Serbia

**Keywords:** High-performance liquid chromatography (HPLC), Charged aerosol detector (CAD), Gradient boosted trees (GBT), Quantitative structure–property relationship modeling (QSPR), Fatty acids

## Abstract

The charged aerosol detector (CAD) is the latest representative of aerosol-based detectors that generate a response independent of the analytes’ chemical structure. This study was aimed at accurately predicting the CAD response of homologous fatty acids under varying experimental conditions. Fatty acids from C12 to C18 were used as model substances due to semivolatile characterics that caused non-uniform CAD behaviour. Considering both experimental conditions and molecular descriptors, a mixed quantitative structure–property relationship (QSPR) modeling was performed using Gradient Boosted Trees (GBT). The ensemble of 10 decisions trees (learning rate set at 0.55, the maximal depth set at 5, and the sample rate set at 1.0) was able to explain approximately 99% (Q^2^: 0.987, RMSE: 0.051) of the observed variance in CAD responses. Validation using an external test compound confirmed the high predictive ability of the model established (R^2^: 0.990, RMSEP: 0.050). With respect to the intrinsic attribute selection strategy, GBT used almost all independent variables during model building. Finally, it attributed the highest importance to the power function value, the flow rate of the mobile phase, evaporation temperature, the content of the organic solvent in the mobile phase and the molecular descriptors such as molecular weight (MW), Radial Distribution Function—080/weighted by mass (RDF080m) and average coefficient of the last eigenvector from distance/detour matrix (Ve2_D/Dt). The identification of the factors most relevant to the CAD responsiveness has contributed to a better understanding of the underlying mechanisms of signal generation. An increased CAD response that was obtained for acetone as organic modifier demonstrated its potential to replace the more expensive and environmentally harmful acetonitrile.

**Supplementary Information:**

The online version contains supplementary material available at 10.1186/s13321-021-00532-0.

## Introduction

Among the various detectors used in high-performance liquid chromatography (HPLC), the UV detector is frequently referred to as the workhorse, being predominantly employed for quality control purposes and routine analysis. Though characterized by high a sensitivity, a broad linear dynamic range, and a user-friendly application, the prerequisite for the usage of the detector is the existence of UV absorbing structural features of the analytes known as chromophores, such as aromatic ring systems or conjugated double bonds. Thus, the detector suffers from poor sensitivity toward analytes lacking suitable chromophores like fatty acids and sugars. This shortcoming can be addressed by using universal detection techniques instead, e.g. aerosol-based detection techniques [1]. The most recent aerosol-based detector, the charged aerosol detector (CAD), stands out in terms of response uniformity due to its unique principle of detection. In contrast to the other aerosol-based detectors, the analyte particles obtained from evaporation of aerosol droplets that were previously generated by nebulization of the mobile phase, are charged by diffusion processes independent of the particle characteristics [2]. In comparison, the refractive index and thus the analyte characteristics comprising the dried particle is critical for the measurement of the light dispersion in evaporative light scattering detection (ELSD). This difference results in higher uniformity in CAD response compared to ELSD [3, 4]. Condensation nucleation light scattering detection (CNLSD) dependence on particle characteristics is even more pronounced, further reducing response uniformity [3, 5]. However, the response of the CAD is not truly uniform, as a mobile phase gradient, the analyte volatility, salt formation, and the analyte density also have an impact on signal generation [2]. Thus, the molecular properties of the analytes as well as the chromatographic conditions must be considered when developing methods to achieve uniform response of the analytes. Several approaches have been reported aimed at generating uniform CAD response, including the application of inverse gradient programs [6] and the establishment of models describing the influence of experimental parameters [7]. However, there is little evidence on the predominant analyte-related and experimental factors influencing CAD response when investigating a set of structurally similar analytes of varying volatility.

**Fig. 1:**
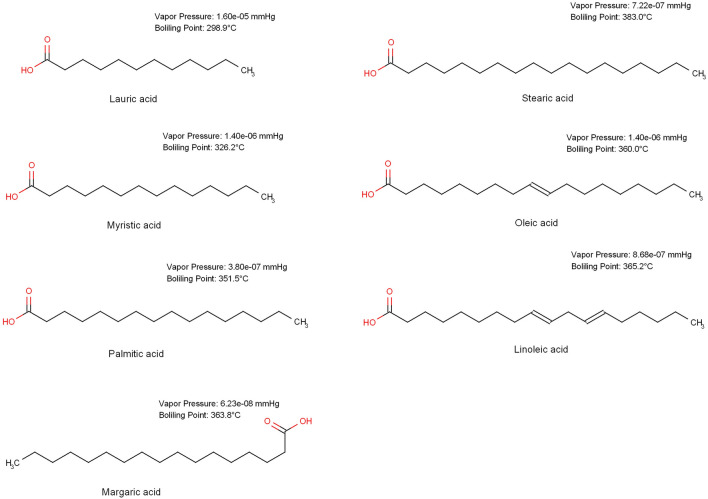
Structural formulas of the seven fatty acids utilized as model substances, with the corresponding vapor pressure and boiling point values

In this study, a homologous series of chromophore-deficient fatty acids (Fig. 1) was selected to evaluate the influence of experimental parameters and molecular properties on the CAD response. Despite their similar structure, the molecular properties of the fatty acids, e.g. the volatility, vary as a function of chain length (Fig. 1). Thus, a comprehensive model accurately describing the influence of the chain length of the fatty acids on the CAD signal would contribute to a better understanding of the underlying mechanisms of signal generation. Such a mathematical tool could reliably estimate the most significant molecular characteristics contributing to the higher CAD responsiveness. To enable drawing valid conclusion on the influence of the experimental and molecular parameters on CAD response of the selected fatty acids, a mixed quantitative structure–property relationship (QSPR) approach was applied. The QSPR model was built with the aid of a Gradient Boosted Trees (GBT) machine learning algorithm (MLA). GBT algorithms combine predictors in a sophisticated manner that can reveal complex patterns that other techniques may miss. GBT utilizes boosting as a technique of building predictive models of elevated complexity that can be superior to other MLAs such as artificial neural networks (ANNs) that were used in similar QSPR studies [8]. Thus, a mixed GBT-QSPR model was employed to accurately describe the influence of experimental parameters and molecular properties on the CAD response. The most significant factors were then evaluated comparing their individual impact on the CAD response. Special emphasis was placed on the environmentally friendly alternatives, acetone and ethanol (EtOH), to the more commonly used organic solvents acetonitrile (ACN) and methanol (MeOH). By validating the effectiveness of green solvents, CAD’s potential to be employed in green chromatography [9] could be demonstrated.

## Materials and methods

### Chemicals and reagents

Lauric acid (98%), linoleic acid (≥ 99%), margaric acid (≥ 98%), myristic acid (≥ 99%), oleic acid (≥ 99%), palmitic acid (≥ 99%), and stearic acid (≥ 98.5%) as well as formic acid (98–100%), HPLC plus grade acetone, HPLC gradient grade acetonitrile (ACN), HPLC grade ethanol (EtOH), and HPLC gradient grade methanol (MeOH) were purchased from Sigma Aldrich (Steinheim, Germany). Ultra-pure deionized (DI) water was delivered by a Milli-Q® system (Merck, Darmstadt, Germany).

### Instrumentation

The experiments were performed on a Thermo Scientific Vanquish™ Flex modular chromatographic system (Thermo Fisher Scientific, Germering, Germany) consisting of a binary pump with online degasser, a thermostatted split sampler, a thermostatted column compartment with passive pre-heater, and a variable wavelength detector in-line with a Vanquish™ Horizon CAD. The CAD was supplied with nitrogen gas from an ESA nitrogen generator (Thermo Fisher Scientific) connected to the in-house compressed air system. The HPLC instrument was controlled and runs were processed using the Chromeleon® Data System Version 7.2.6 software program (Thermo Fisher Scientific).

### Preparation of solutions

Stock solutions of the fatty acid standards were prepared by accurately weighing 5.0 mg of the respective fatty acid and dissolving in acetone, ACN, EtOH, and MeOH, respectively. The stock solutions were diluted to a concentration of 50 µg/mL with a mixture of DI water and organic solvent in proportions equivalent to the mobile phase composition used according to the experimental plan in each case (Additional file 1: Table S1, hosted by figshare [10]).

### Flow injection analysis (FIA)

The outlet capillary of the Vanquish™ sytem’s injection valve was linked to the inlet capillary of the UV detector by a connector (Viper™ union, Thermo Fisher Scientific) to perform the FIA at sufficient back pressure. Isocratic runs with a runtime of 2 min, an injection volume of 10 µl, and flow rates of 0.5 mL/min, 1.0 mL/min, and 1.5 mL/min, respectively, were carried out. The temperature of the column chamber was held constant at 25 °C, while the mobile phase consisted of 0.1% (v/v) formic acid in DI water and 0.1% (v/v) formic acid in either acetone, ACN, MeOH, or EtOH in various proportions (75%, 82.5%, 90% (v/v) organic solvent) according to the experimental plan (Additional file 1: Table S1, hosted by figshare [10]. Prior to each new run, the system was equilibrated for 5 min at the upcoming mobile phase conditions. When switching the organic solvent, the system was flushed for at least 30 min at the upcoming mobile phase conditions until a stable baseline was obtained. CAD was employed for the detection of the fatty acids with the instrumental settings evaporation temperature (24 °C, 36 °C, 48 °C) and power function value (PFV) (0.8, 1.2, 1.6) being altered according to the experimental plan (Additional file 1: Table S1 [10]), whereas the filter constant was maintained at 1.0 s.

### Selection of the experimental variables and their design levels

To evaluate the influence of various experimental parameters on the CAD response, screening experiments by FIA based on a ${2}^{5-1}$ fractional factorial design (FFD) were performed. The selected factors and their corresponding ranges were as follows: the organic solvent’s content in the mobile phase (70–90 $\%$, v/v), the flow rate of the mobile phase (0.5–1.5 mL/min), the evaporation temperature (24–48 °C), the PFV (0.8–1.6), and the filter constant (1–5 s). The FIA experiments were conducted with four different organic solvents, namely ACN, MeOH, acetone and EtOH. In order to estimate the experimental error, 4 additional runs at the central point of the experimental domain were included in the FFD plan. With lauric acid, palmitic acid, and stearic acid representing the low, medium, and high levels of the set of analytes investigated, 20 experiments with 4 different solvents were performed in a randomized order for each fatty acid. The significance of the examined factors’ influence was assessed using Student’s t-tests and Pareto diagrams.

Response surface methodology (RSM) was subsequently employed for the thorough description of the experimental domain. The selection of parameters investigated with their respective ranges was based on the results of the screening phase, except for the content of the organic solvents. Their low levels were increased from 70 to 75% v/v, respectively, due to the insufficient solubility of stearic acid in higher aqueous proportion. The statistically significant factors derived from the screening experiments were varied according to the experimental plan created by Central Composite Design (CCD). Within the experimental plan, the type of the organic solvent used was coded by assigning the numbers 1–4 to ACN, MeOH, acetone and EtOH, respectively. The plan of the CCD is depicted in Additional file 1: Table S1 [10]. The filter constant was maintained at 1 s, since it did not significantly influence the CAD response. The RSM experiments were carried out in random order. The magnitude of the CAD response was studied as the system’s response.

Design-Expert 7.0.0. (Stat-Ease, Inc., Minneapolis, USA) was used to construct the ${2}^{5-1}$ FFD and the CCD experimental plans.

### Computation of the molecular descriptors

The chemical structures of lauric acid, myristic acid, palmitic acid, margaric acid, stearic acid, oleic acid, and linoleic acid were sketched in ChemDraw Ultra 8.0 software (PerkinElmer, Massachusetts, USA). Each structure was subjected to geometry optimization using the semi-empirical MOPAC/PM3 method in Chem 3D® Ultra 8.0 (Cambridge Soft Corporation, Cambridge, USA). The compounds’ conformations with the minimum energy were used to calculate physico-chemical, topological, geometrical, and spatial structural descriptors in Dragon 6.0.7. software (Talete srl, Milano, Italy). To prevent potential correlation issues, descriptors that were strongly correlated to the other descriptors (using a correlation coefficient │r│ > 0.90), descriptors with constant values (RSD < 5%), i.e. descriptors that were not available for all analytes were excluded. After this step, the set of several thousand descriptors originally calculated was reduced to 60 molecular descriptors.

### Exploratory analyses

Basic statistics (mean, min, max, etc.) of each feature are described in Additional file 2: Table S2. The range (max–min) for the experimental factors was chosen to ensure the satisfactory CAD response of the tested compounds. Due to the DoE approach used and symmetrical placement of − 1 and + 1 levels around nominal (0) level, the mean and median were exactly the same for these attributes. As for other attributes (molecular descriptors), descriptive statistics was determined by the structure of the analytes. For instance, the higher representation of C18 than C12–C13 fatty acids in the dataset caused the MW descriptor to have a greater median than the mean. In the same way, other reported statistics was as expected. Also, no missing data were observed.

### Calculation of the skewness coefficients

Skewness coefficients were obtained from the SKEW function in Excel 2010 (Microsoft Office, Redmond, Washington, USA). By the means of the SKEW function, skewness ${G}_{1}$ of sample $S$ containing $n$ number of random variables $x$ is estimated as follows (Eq. 1):

1 $${G}_{1}=\frac{n}{(n-1)(n-2)}\sum {(\frac{{x}_{i}- \overline{x}}{s })}^{3}$$

In Eq. 1, $s$ is the standard deviation of a data set S, while $\overline{x }$ is the mean. It should be applied only if $n$ >2. The skewness coefficients were calculated separately for the training and the test data.

### GBT algorithm

Decision tree (DT) is a machine learning algorithm that splits a feature space by which objects are described, into several different and mutually excluded subspaces by a recursive partitioning method [11, 12]. It is usual accompanied by a tree-like diagram that displays different outcomes from a series of decisions. Among the available range of techniques utilized for real-world data, DT is favored for its easiness of interpretation and elegant ability to work with missing values [13–15]. Additionally, DT is capable of dealing with extensive datasets and neglecting redundant descriptors, which makes it quite useful in QSPR model building [16].

On the other hand, DTs are characterized as weak learners. Additionally, even a slight change within the training set could lead to a major change in the algorithm topology, making DTs unstable classifiers. Therefore, a concept of building additive tree structures based on ensemble learning has been adopted [16, 17]. Ensembles that probably achieve better predictive performance than individual constituent (base) algorithms are typically generated by using boosting and bagging techniques. GBT utilize boosting as a technique of building predictive models of elevated complexity. Boosting is regarded as one of the most powerful ideas introduced in the last few decades within the machine learning domain [18]. Within particular concept, the individual (base) algorithms are combined in a sequence in order to provide a solution to a demanding computational problem. Prevention of mutual correlations between trees, induced by the engagement of the same training set, is managed using certain “penalties”. These penalties, by repeatedly modifying original data, put emphasis on the errors made by the previous algorithms and, consequently, facilitate the process of learning for the currently added tree. At each step, the model employs the algorithm that best fits the current residuals. This process is usually repeated many times. During each step, parameters of existing trees are kept unchanged, giving rise to so-called stage-wise additive modeling. The purpose of this approach is to reduce the risk of overfitting.

In order to detect the residuals, a loss function is used. GBT sequentially combines DTs in way that each new added instance minimizes arbitrarily chosen, differentiable loss functions in descent gradient fashion. In terms of mathematical principles, GBT model can be presented using Eq. 2:

2 $${f}_{i}\left(x\right)={f}_{i-1}\left(x\right)+\nu {w}_{i}{G}_{i}\left(x\right);\,0<\nu \le 1$$

In the Eq. 2 $fi(x)$ and ${f}_{i-1}(x)$  are models constructed at iteration $i$ and $i-1$, respectively. The term denoted as ${w}_{i}$ represents weight (“penalty”) while $\nu$ is a regularization parameter—shrinkage or learning rate. The lower the learning rate, the slower the model learns. At the same time, it achieves better performance in terms of accuracy. However, if the learning rate is low, more trees are needed to be included in the ensemble. Engagement of too many trees indicates a high risk of overfitting. The identification of ${G}_{i}(x)$ required to be added to the model is the primary optimization problem.

### Predictive modeling workflow

A QSPR modeling workflow was created using the Rapidminer Studio 9.1.000 (RapidMiner, Boston, MA, USA) software. The data related to margaric acid were excluded from the primary set and used as external test set. The remaining data were divided into 10 subsets of equal size by the Cross Validation Operator. This is a nested Operator that has two subprocesses. Inside the first subprocess of the Cross Validation Operator, the GBT algorithm was trained on 9 of the 10 subsets. The trained model was then applied in the second subprocess where its performance was measured. The omitted subset was used as an input of the testing stage. This procedure was repeated 10 times, so that each subset was used one time as a test set. The overall model’s performance was estimated by averaging the results (cross-validation correlation coefficient, Q^2^ and root mean squared error, RMSE) from 10 iterations. The subsets used in the Cross Validation procedure were made by shuffled sampling.

After being trained and tested, the GBT-based QSPR model was applied to the external validation test set of the margaric acid data using the Apply model operator. The actual predictive power of a given model was quantified in terms of root mean squared error of prediction (RMSEP) and R^2^. The RMSEP and R^2^ estimates were obtained from the Performance operator. The detailed Rapidminer workflow is available as part of the Additional file 1. The optimal performance of the model was achieved by grid tuning of hyperparameters, namely the learning rate (0.1–1.0, 30 steps); maximum depth (5–10, 6 steps) and sample rate (0.1–1, 9 steps). The number of decision trees (4–20, 9 steps) was adjusted by a trial-and-error approach. The number of trees was chosen to prevent overfitting. All hyperparamaters were adjusted to reduce the RMSEP.

## Results and discussion

### Selection of the fatty acids investigated

**Fig. 2:**
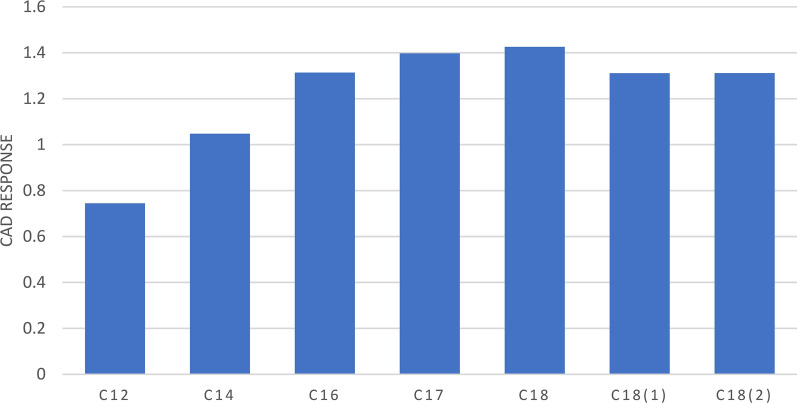
Average CAD response for the fatty acids investigated obtained from FIA. The fatty acids are coded with their respective number of C-atoms. The degree of unsaturation is indicated in brackets where applicable

The uniform response of the CAD for non-volatile analytes has been demonstrated in multiple studies [7, 19, 20]. Thus, the selection of semi-volatile and non-volatile fatty acids that are structural homologues but differ significantly in their response was essential to develop a model that could accurately predict the CAD response based on a mixed model including the response-determining molecular descriptors. Previous studies on the CAD response of fatty acids revealed a pronounced decline in the response of myristic acid (C14) compared to its structural homologue palmitic acid (C16) [21]. With respect to this preliminary observation, fatty acids ranging from lauric acid (C12) to stearic acid (C18) were selected as test substances due to their estimated differences in CAD response. The differences in response were evident when comparing the average response values obtained for each fatty acid from the CCD based FIA runs as depicted in Additional file 1: Table S1 [10]. Going from lauric acid (C12) to stearic acid (C18), the CAD response increased with the chain length of the fatty acids (Fig. 2). Interestingly, a pronounced decline in response could be observed between myristic acid (C14) and palmitic acid (C16), while the response for fatty acids > C16 did not significantly increase. Thus, fatty acids < C16 can be considered as semi-volatile compounds. These results strongly indicated the need for a mixed model as was employed here to include the molecular properties of the fatty acids in the modeling of the CAD response.

### DoE assisted development of the QSPR dataset

Design of experiments (DoE) is used to test versatile hypotheses in efficient manner. Within the DoE concept, independent variables (factors) are systematically varied in order to draw conclusion about factors’ influences toward the target response [22]. In this study, DoE was utilized with a two-fold purpose. To examine the impact of the five pre-selected experimental factors and their possible interactions on the response of interest using DoE, it was necessary to conduct 32 (2^5^) screening experiments. However, with the general goal of preserving all possible resources in the first phase, it was decided to fractionalize the experimental plan. Application of FFD allowed valid conclusions to be drawn with only 16 experiments per 4 different organic solvents. In the screening stage, the CAD responses were measured for three fatty acids (lauric acid, palmitic acid, and stearic acid). In accordance with their structural characteristics, lauric, palmitic and stearic acid were the representatives of the tested compounds. Therefore, in dependence of the fatty acid’s chain length, estimated CAD responses could be at low, medium, or high level. According to the applied tests, the most significant experimental parameters toward CAD response were: the type of the organic solvent, its proportion in the mobile phase, the flow rate of the mobile phase, the evaporation temperature, and the PFV.

The usage of ${2}^{5-1}$ FFD enabled a preliminary assessment of the QSPR model’s performance that was subsequently conducted. Namely, if no screening has been carried out and an insignificant experimental variable (filter constant) had been included in the model development, the GBT algorithm would have been loaded with identical cases in the training phase. The use of identical cases (that is, examples that differ in the values of insignificant factor) in the learning stage would likely lead to an overestimation of the QSPR model’s predictive ability.

In the following CCD experiments, the examined levels of significant factors were retained from the screening stage along with the obligatory addition of a central level. As an exception, the low value of the organic solvents’ volume fraction was increased from 70 to 75% (v/v) due to the precipitation of stearic acid at 70% MeOH (v/v) proportion. The insignificant parameter filter constant was maintained at 1 s, since it was associated with the lowest background noise. The CCD experiments were carried out in random order to minimize the effects of uncontrollable variables.

Significant factors and a combination of their values according to CCD are incorporated in Additional file 1: Table S1 [10], together with the results of the experiments.

### Distribution of the outcome variable

Prior to the statistical analysis, the distribution of the experimentally obtained CAD responses was examined. Therefore, the skewness coefficient of the distribution was calculated in accordance with Eq. 1. In numerical terms, the skewness of a normal distribution is approximately zero. If the given coefficient is less than − 1 or more than + 1, the distribution is highly skewed, while the distribution is moderately skewed if the coefficient is between − 1 and − 1/2 or between + 1/2 and + 1. Positive coefficients indicate positive skewness and vice versa [23, 24].

From the machine learning perspective, a highly skewed distribution could impair the predictive performance of the models developed. This claim finds its support in the fact that machine learning algorithms try to minimize the prediction error by learning to predict the response in the densest region of endpoints. As an implication of this concept, it is less likely that these algorithms will successfully predict the response of those endpoints that do not reside in the densest area. The usual strategy for addressing this issue is the transformation of the skewed variable, that is, the application of the same function to each of its values [25].

Here, the target variable showed a highly skewed distribution with a skewness coefficient of + 1.8 calculated from Eq. 1 (Additional file 2: Fig. S1a). Common transformations applied to positively skewed data include logarithmic, square-root, and cube-root transformation [25–29]. Given how logarithmic, square-root, and cube-root transformed data displayed a skew of − 0.70, 0.79, and 0.36, respectively, it was decided to use the latter transformation in the QSPR model construction. Additional file 2: Fig. S1b shows the distribution of the target response after applying the cube root transformation to each value.

### QSPR modeling

The aim of this study was to develop a QSPR model that could predict the CAD response of fatty acids showing different volatility in a certain experimental domain with satisfying accuracy. In addition, the identification of the most important experimental and response-determining structural features would contribute to a comprehensive and mechanistic understanding of signal generation.

The QSPR model was built by linking the molecular descriptors computed for 6 fatty acids representing semi-volatile and non-volatile compounds to their CAD responses via GBT. The responses were measured under 25 different experimental conditions for each of the four organic solvents. As stated in "Distribution of the outcome variable", the output values were transformed using the cube-root (see Additional file 1: Table S1 [10]) to remove the skewness from the experimental data.

To demonstrate the validity of the applied modeling approach, a conventional QSPR model and a RSM model that solely linked the experimental parameters to the transformed CAD responses were developed simultaneously. The competing models, however, showed poor predictive performance compared to established mixed QSPR model. These results supported the assumption that the CAD response depends on both the experimental parameters and the molecular properties of the tested compounds. Hence, only the inclusion of both independent variables in the model provided a large rate of explained variance as well as enough observations that could be used in the process of training a machine-learning algorithm [8, 30].

However, before any model can be used in practice, the reliability of its application must be confirmed by different validation procedures [31]. In this study, the GBT-based mixed model was validated via tenfold cross-validation and, in addition, by an external validation set. The predictive ability of the developed QSPR relationships was evaluated using data related to margaric acid, which were not employed in the model generation. The test analyte was chosen with respect to its structural properties at the intermediate level of the fatty acids investigated and due to its similar distribution of CAD responses compared to the training set. The obtained responses for margaric acid were also included in Additional file 1: Table S1 [10].

GBT was used as model building technique due to beneficial intrinsic attribute selection strategy and a pronounced ability to predict a target value that was close to the true response value for the given observations. The GBT-based QSPR model with the learning rate set at 0.55, the maximal depth set at 5, and the sample rate set at 1.0, showed satisfactory performance in terms of low RMSE, i.e. RMSEP and high Q^2^, i.e. R^2^ values. By using the significant experimental parameters and descriptors listed in "Significant features"., the ensemble of 10 DTs was capable to explain approximately 99% (Q^2^: 0.987, RMSE: 0.051) of the observed variance in CAD responses. Low RMSE values of 0.050 and high R^2^ values of 0.990 for the external validation set suggested the high predictive ability of the model developed. The consistency of the tenfold CV with the external validation results clearly indicated that no overfitting occurred in the learning stage. The performance statistics are summarized in Additional file 2: Table S3.

**Fig. 3:**
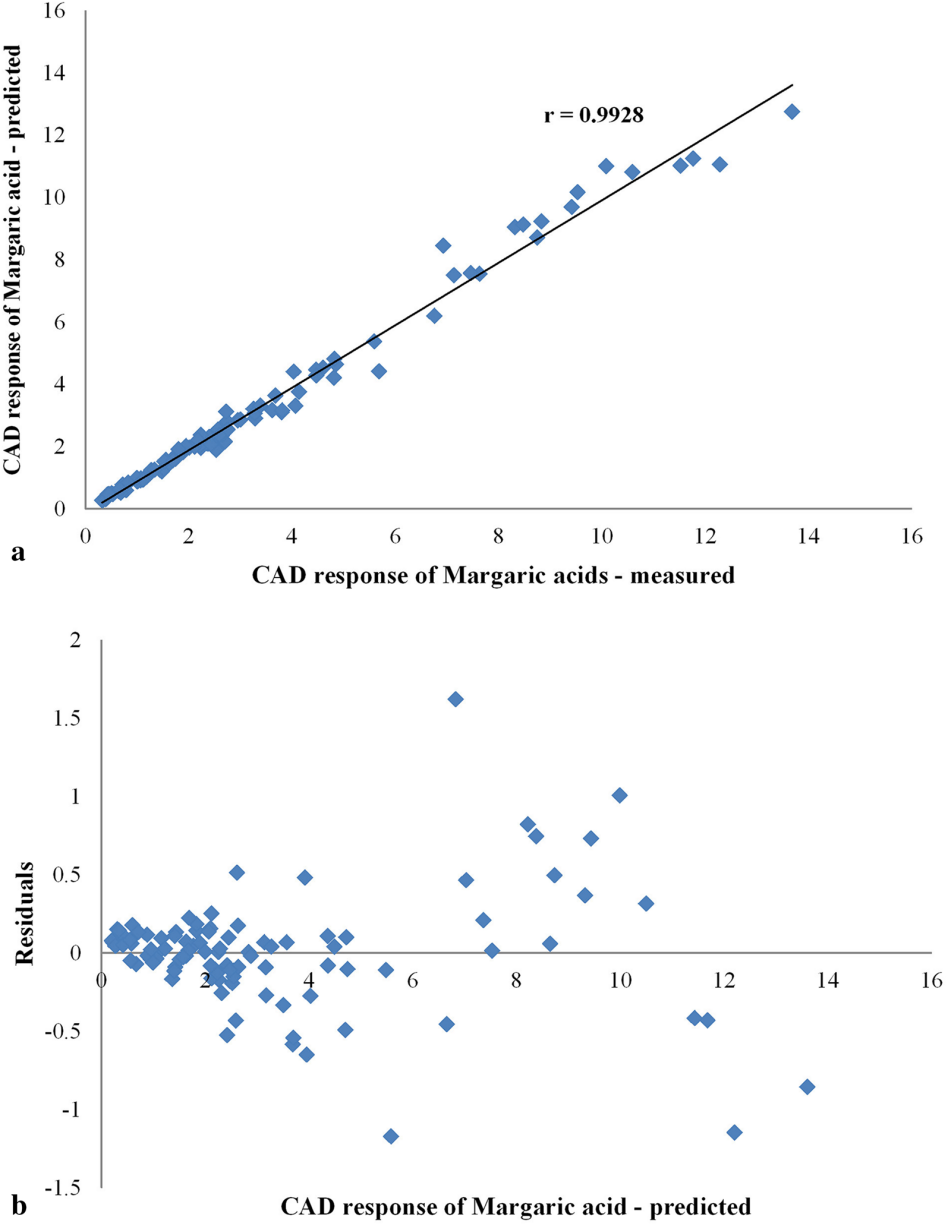
**a** Regression plot of the optimized GBT-QSPR model. **b** Residual plot of the optimized GBT-QSPR model

Considering that the QSPR model development involved cube-root transformed output data, it was decided to examine whether the model’s performance was retained for the back transformed response values. In this regard, the correlation between the measured and the predicted CAD responses for the validation data set is visualized and presented in Fig. 3a. It can be noticed that there are few endpoints (with values of CAD response between 6 and 14) that have been poorly predicted by the GBT-QSPR model. It is possible that an estimation error occurred due to the utilized set of attributes. In other words, a different set of input variables might be able to better distinguish responses within the given range. It is equally probable that GBT did not show the best adaptation to the generated data and that some other machine learning algorithms could more accurately learn the patterns contained in the experimental results. Nonetheless, a comprehensive analysis of the observed phenomenon is going to be the subject of prospective studies.

To provide a more detailed discussion on the predictive performance of the developed model, the residuals of GBT regression were visually inspected. The residual plot (Fig. 3b) shows the distribution of overestimated and underestimated CAD responses. Ideally, all residuals should be small; this would indicate reasonable underlying assumptions and appropriateness of the fitted model [32]. As can be seen, the predictions met this criterion for low CAD response values (up to 4). However, as the fitted values increase, the residuals tend to deviate more from the 0% error line. Due to the investigated ranges of experimental variables, and, consequently, a much smaller number of observations with larger values of CAD response, this result was somewhat expected.

### Significant features

In general, the model developed by GBT found non-linear patterns of molecular descriptors and experimental parameters that predicted the CAD response of the fatty acids investigated relatively well. However, the provided accuracy came at the cost of low interpretability. In order to address this issue, the variable importance tool was used.

**Fig. 4:**
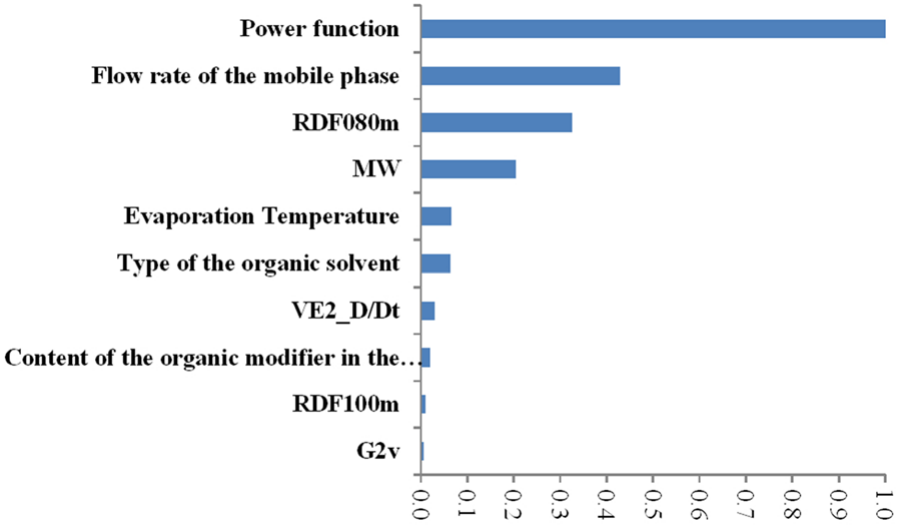
The independent variables (y-axis) and their importance (x-axis) toward CAD response

With the intrinsic strategy of attribute selection, the GBT algorithm makes use of all independent variables available while forming the model [33]. The attributes with the highest scaled importance were considered as most relevant toward the CAD response of the fatty acids investigated. The ten attributes (y-axis) with the highest scaled importance (x-axis) in descending order are shown in Fig. 4. As it can be seen, GBT assigned great importance to the PFV, the flow rate of the mobile phase, and the molecular descriptors molecular weight (MW), Radial Distribution Function—080 / weighted by mass (RDF080m) and average coefficient of the last eigenvector from distance/detour matrix (Ve2_D/Dt). It should be noted that the signal generation of the CAD was also influenced by the CAD’s evaporation temperature and the proportion of the organic solvent in the mobile phase, but to a much lesser extent. The impact of the significant factors on the CAD response is addressed thoroughly below, including graphical representations of the found patterns. The graphs provided are also part of a strategy to increase the interpretability of the GBT-based model.

**Fig. 5:**
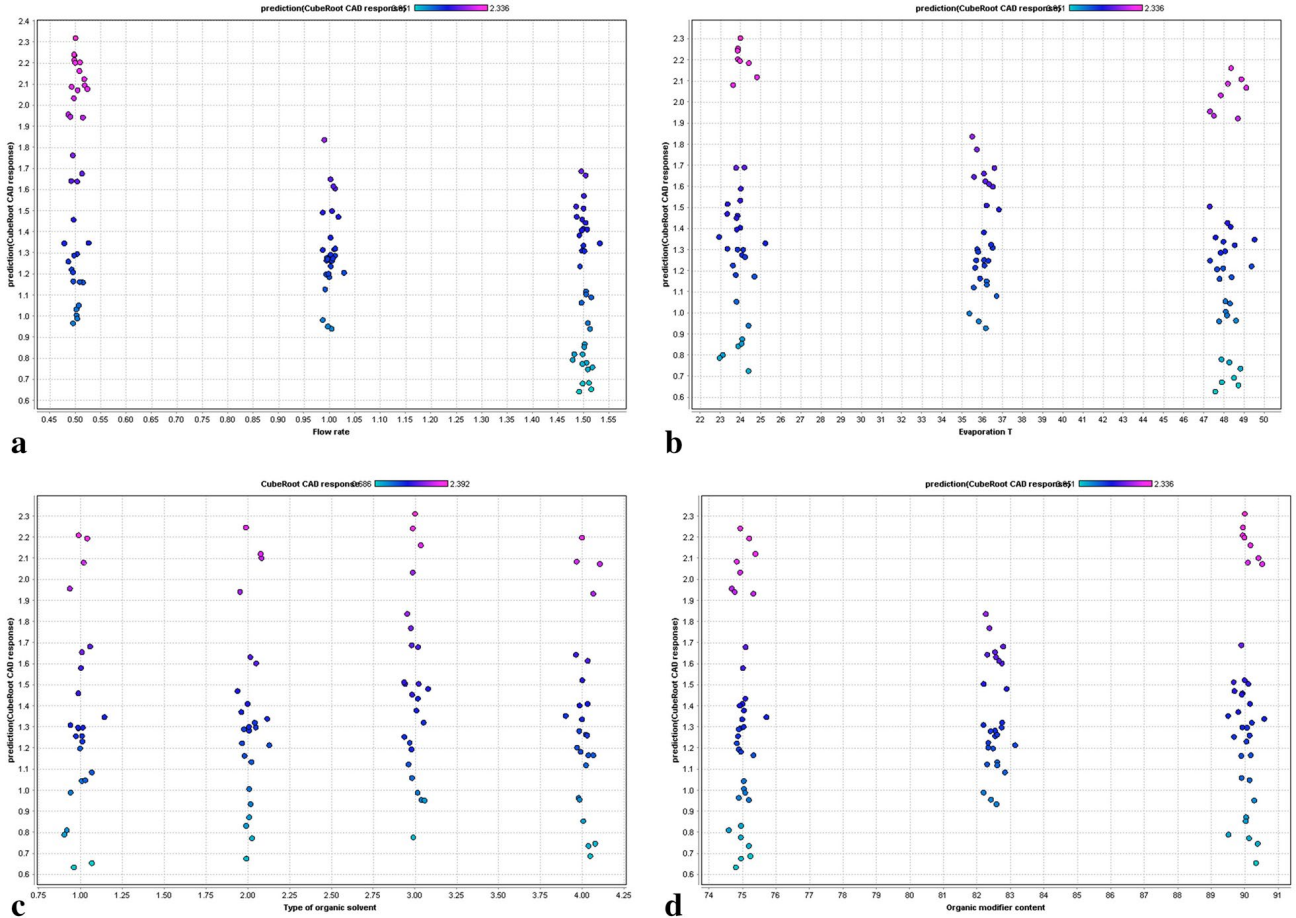
Graphs showing the relationships between the predicted CAD response and **a** flow rate, **b** evaporation temperature, **c** type of organic solvent: 1—ACN; 2—MeOH; 3—Acetone; 4—EtOH, **d** the content of organic solvent in the mobile phase (v/v)

The PFV setting raises the CAD’s raw signal current to a specified power, thereby altering the signal output of the detector [2, 34, 35]. PFV other than the default value of 1.0 can be applied to improve the detector’s linearity in the range of interest [2]. Therefore, its influence on the CAD’s response is evident (Additional file 2: Fig. S2), as the signal is directly modified by the respective PFV, which could be confirmed by its scaled importance value of 1.0. Thus, despite no additional information on the underlying mechanisms of signal generation was obtained here, the result supports the validity of the applied model. The influence of the flow rate being the most important among the chromatographic parameters toward CAD response was in line with the principle of function of the detector. Low mobile phase flow rates produce initial droplets of smaller size; thus, their evaporation is sped-up compared to larger droplets, which is beneficial for the subsequent detection process [36, 37]. Consequently, the CAD response of all fatty acids examined increased with flow rate reduction (Fig. 5a). Besides the two most influential parameters, the evaporation temperature, as one of the adjustable CAD settings, also had an impact on the CAD response. The evaporation temperature setting can be altered to adjust the selectivity of the detector toward a certain analyte dependent on its volatility [2]. Hence, low evaporation temperatures lead to a broader detection range due to the improved detection of semi-volatile analytes. The optimal evaporation temperature for a certain analyte requires experimental optimization, as the background noise of the CAD also changes with evaporation temperature due to enhanced evaporation of mobile phase impurities. However, at higher evaporation temperatures, analytes with semi-volatile characteristics, such as the short/medium chain fatty acids lauric acid and myristic acid, are expected to suffer a substantial loss of response [21]. Therefore, lower evaporation temperatures are contributing to enhanced CAD response for these fatty acids, and, consequently, could result in improved S/N (Fig. 5b).

Among the organic solvents commonly used with the CAD, ACN and MeOH are the predominantly applied solvents. The properties of the organic solvents applicable to the CAD, e.g. their low surface tension and viscosity, are beneficial for enhancing the detector’s response due to the more efficient nebulization and aerosol transport processes compared to aqueous solvent [2]. In the current study, four different solvents were investigated, namely ACN, MeOH, acetone and EtOH. Figure 5c illustrates that there were no remarkable differences toward CAD response obtained with the different solvents. However, slightly higher CAD responses were obtained with acetone in comparison to the remaining solvents. This observation is in accordance with the properties of the organic modifier, since acetone has the lowest viscosity and highest vapor pressure among the organic solvents investigated, which promotes efficient nebulization and evaporation. The use of inexpensive and environmental friendly acetone in experiments with CAD (instead of ACN) was previously suggested by Hutchinson et al. [38]. Apart from the response-enhancing properties of the solvent itself, the content of the organic solvent in the mobile phase also influences the magnitude of response generation. The organic solvent content in the mobile phase was varied in a rather small range from 75 to 90% (v/v) in our experiments due to solubility issues of some fatty acids at higher aqueous proportions. In addition, the separation of fatty acids is often achieved using mobile phases with high organic contents on C18 stationary phases [21, 39]. The CAD response did not notably change with organic solvent content, which was somewhat expected. Slightly higher CAD responses were obtained with 90% (v/v) of organic solvent in the mobile phase (Fig. 5d), but the investigated range was too narrow for significant results. However, the influence of the organic content on CAD response has been evaluated in multiple studies and can therefore be regarded as evident [37, 38, 40]. It has to be kept in mind, that the variation of the organic content in the mobile phase is limited due to the separation of analytes.

A significant feature of the so-called universal detectors that justifies their use instead of the commonly applied UV detector, is the minor influence of the physicochemical properties of the analytes on the response. In fact, a relatively uniform response for non-volatile analytes has been demonstrated for the CAD in numerous studies [7, 19, 20]. However, there might be analyte-related properties influencing the CAD’s response at constant experimental settings, such as the density of the compounds, their charge, the hydrogen bond donor capability [41], and the number of electronegative atoms [8]. Consistent with these assumptions, the results of the study performed indicate that there are indeed certain molecular properties influencing the CAD responsiveness.

**Fig. 6:**
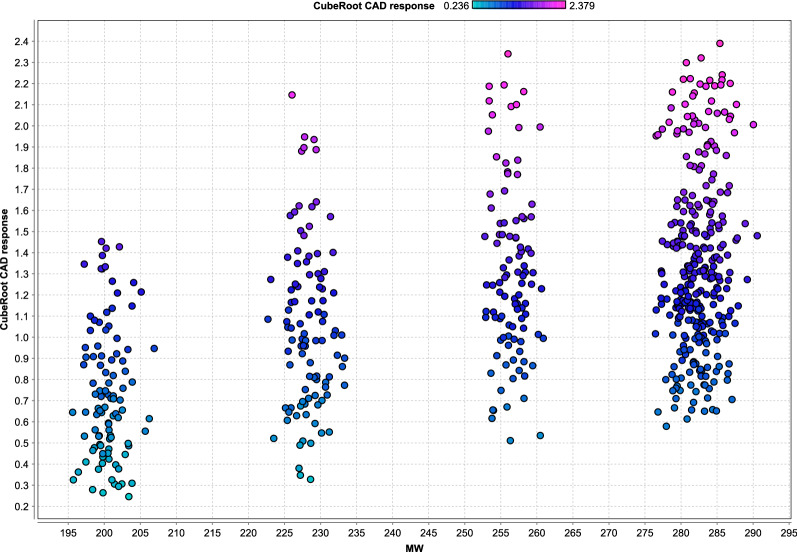
Effect of the MW of the fatty acids investigated on the CAD response

Among the molecular properties that may have an impact on the response of aerosol-based detectors, the volatility of a compound, which is often characterized by its vapor pressure or boiling point, can be regarded as the most significant property toward detector responsiveness due to the mandatory evaporation step in the detection process of all aerosol-based detectors. The volatility of an analyte is strongly affected by its molecular weight [2]. Thus, the great importance attached to the MW descriptor by GBT is in accordance with the volatility requirements as stated above. However, there are no distinct limits determining the analyte as volatile, and, additionally, the volatility also depends on the experimental conditions, such as the CAD’s evaporation temperature or the formation of less volatile salts with mobile phase additives [2]. While the response for analytes with low molecular weight tends to be decreased and non-uniform due to their relatively high volatility, the response for analytes with a molecular mass > 300 Da can be regarded as independent of volatility and more uniform [42]. Figure 6 confirms the positive correlation between molecular weight and CAD response, in case of the homologous fatty acids. As illustrated, the CAD response increases with chain length and thus molecular weight of the fatty acids.

**Fig. 7:**
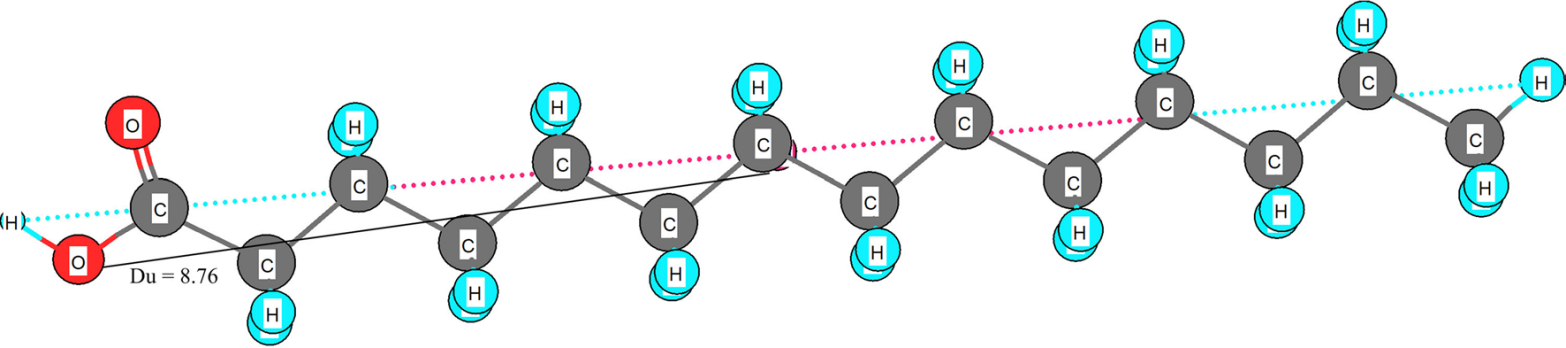
Geometrical center of the 3D represented myristic acid

RDF080m, the most significant among the utilized descriptors, belongs to the class of Radial Distribution Function (RDF) molecular descriptors. It represents a three-dimensional mass distribution calculated at a radius of 8 Å from the center of a geometrical representation of the molecule. The high importance of this descriptor points out a high contribution of steric factors at the radius of 8 Å from the molecules’ geometrical center to the observed response [43]. The different distance of the carboxyl groups from the geometrical center of the molecules investigated is most likely responsible for the distinct differences in CAD response corresponding to the chain length of the fatty acids. The respective distance of the carboxylic group from the geometric center of the fatty acids ranges from 4.77 to 10.52 Å. Stearic acid (C18) with a carboxyl group furthest from the geometric center showed the largest CAD response. It is followed by the response of margaric (C17, 9.36 Å) and palmitic acid (C16, 9.28 Å). The geometric center and its distance (Du) from the carboxyl group are shown exemplarily for myristic acid (C14) in Fig. 7.

Ve2_D/Dt is categorized as a 2D matrix-based molecular descriptor. Here, it specifically defines the average coefficient of the last eigenvector from a distance/detour matrix. Basically, it indicates that the topological distribution of molecular charge and mass might have some impact on CAD responsiveness [44]. This descriptor negatively affects the intensity of the CAD response, according to graph constructed via GBT (Additional file 2: Fig. S3).

## Conclusions

The influence of the molecular properties and experimental conditions on the observed CAD response was investigated for a homologous series of fatty acids of varying volatility using a GBT-QSPR approach. The applicability of the QSPR patterns was studied in a 5-dimensional experimental space, comprising PFV (0.8–1.6), evaporation temperature (24–48 $^\circ{\rm C}$), flow rate of the mobile phase (0.5–1.5 mL/min), organic solvent (ACN, MeOH, EtOH, acetone), and content of organic solvent in the mobile phase (70–90 $\%$, v/v).

The reliability of the mixed QSPR model was confirmed by the tenfold cross-validation and the external validation. The established pattern could explain 99% (Q^2^: 0.987, RMSE: 0.051) of the observed variations in CAD responses despite the fatty acids’ significant differences in volatility, and, thus, response. Low RMSEP values of 0.050 and high R^2^ values of 0.990 for the external validation set confirmed that the developed model was capable to predict the CAD response for previously untested structural homologues with satisfying accuracy.

Though the CAD is often referred to as a detector producing a uniform response, the successfully established mixed model revealed the significance of MW, RDF080m and Ve2_D/Dt molecular descriptors toward the signal’s magnitude. The joint importance of molecular weight and evaporation temperature highlighted the dependence of the CAD response on the volatility of the respective analyte. The high impact assigned to the RDF080m descriptor pointed out a significant contribution of steric factors to the generated response. Due to the importance of the Ve2_D/Dt descriptor, the different CAD response for the fatty acids can be partially assigned to versatile topological distribution of charge and mass.

The dependence of the CAD response on the operating conditions was once again confirmed. Thus, an advanced optimization of the corresponding parameters, such as evaporation temperature and flow rate, is highly recommended. Due to the slightly higher CAD responses obtained with acetone in comparison to the ACN, MeOH, and EtOH, its usage in CAD methods could be promising. However, as the elution strength and the background noise also differ among various organic modifiers, more detailed studies concentrated on method development are required to make valid conclusions.

## Supplementary Information

**Additional file 1: Table S1.** Data table for QSPR model building.

**Additional file 2: Table S2.** Basic statistics of employed features.** Table S3.** The results of 10-fold CV and external validation.** Fig. S1.** Distribution of dependent variable, a before cube-root transformation; b after cube-root transformation.** Fig. S2.** Graph showing the relationships between the predicted CAD response and the PFV.** Fig. S3.** Graph showing the relationships between the CAD response of fatty acids and the values of Ve2_D/Dt molecular descriptor.

## Data Availability

All data generated or analysed during this study are included in this article and its supplementary information files.

## Abbreviations

CAD: Charged aerosol detector
CCD: Central composite design
DoE: Design of experiments
DT: Decision tree
FFD: Fractional factorial design
FIA: Flow injection analysis
GBT: Gradient boosted trees
MLA: Machine learning algorithm
PFV: Power function value
QSPR: Quantitative structure–property relationship modeling
