# Supplementary material for: Charged aerosol detector response modeling for fatty acids based on experimental settings and molecular features: a machine learning approach
Source: J Cheminform. 2021 Jul 15;13:53. doi: 10.1186/s13321-021-00532-0 (PMC8281619; doi:10.1186/s13321-021-00532-0)
Supplement: Supplementary file 2 — Additional file 2: Table S2. Basic statistics of employed features. Table S3. The results of 10-fold CV and external validation. Fig. S1. Distribution of dependent variable, a before cube-root transformation; b after cube-root transformation. Fig. S2. Graph showing the relationships between the predicted CAD response and the PFV. Fig. S3. Graph showing the relationships between the CAD response of fatty acids and the values of Ve2_D/Dt molecular descriptor. [file 13321_2021_532_MOESM2_ESM.docx]

**Charged Aerosol Detector response modeling for fatty acids based on experimental settings and molecular features: a machine learning approach**

**Supplemental Material**

Ruben Pawellek^a, 1^, Jovana Krmar^b, 1^, Adrian Leistner^a^, Nevena Djajić^b^, Biljana Otašević^b^, Ana Protić^b*^, Ulrike Holzgrabe^a*^

**^a^** University of Würzburg, Institute for Pharmacy and Food Chemistry, Am Hubland, 97074 Würzburg, Germany

**^b^** University of Belgrade, Faculty of Pharmacy, Department of Drug Analysis, Vojvode Stepe 450, 11 221 Belgrade, Serbia

^1^ These authors contributed equally to this work.

**^*^** Corresponding authors:

Prof. Dr. Ulrike Holzgrabe (Chromatography) 
Institute for Pharmacy and Food Chemistry 
University of Würzburg 
Am Hubland 
97074 Würzburg 
Phone: +49 931 31 85460. 
E-mail address: ulrike.holzgrabe@uni-wuerzburg.de

Assistant Professor Ana Protić (Modeling)

Faculty of Pharmacy 
University of Belgrade 
Vojvode Stepe 450 
11 221 Belgrade 
Phone: +38 111 395 1334. 
E-mail address: ana.protic@pharmacy.bg.ac.rs

Table of content:

Table S1. Data table for QSPR model building (additional file)

Table S2. Basic statistics of employed features (p. 2)

Table S3. The results of 10-fold CV and external validation (p. 5)

Fig. S1. Distribution of dependent variable (p. 6)

1. before cube-root transformation
2. after cube-root transformation

Fig. S2. Graph showing the relationships between the predicted CAD response and the PFV (p. 7)

Fig. S3. Graph showing the relationships between the CAD response of fatty acids and the values of Ve2_D/Dt molecular descriptor (p. 8)

**Table S1. Data table for QSPR model building**

Table S1. is provided as additional file.

| **Table S2. Basic statistics of employed features** | | | | | | |
| --- | --- | --- | --- | --- | --- | --- |
| **Feature/statistics** | **Min** | **Max** | **Mean** | **Median** | **First Quartile** | **Third Quartile** |
| **Organic modifier content** | 75.000 | 90.000 | 82.500 | 82.500 | 75.000 | 90.000 |
| **Flow rate** | 0.500 | 1.500 | 1.000 | 1.000 | 0.500 | 1.500 |
| **Evaporation T** | 24.000 | 48.000 | 36.000 | 36.000 | 24.000 | 48.000 |
| **Power f** | 0.800 | 1.600 | 1.200 | 1.200 | 0.800 | 1.600 |
| **MW** | 200.360 | 284.540 | 255.470 | 268.490 | 228.420 | 282.520 |
| **TIC4** | 146.692 | 245.668 | 191.741 | 178.274 | 160.547 | 240.989 |
| **VE2_D/Dt** | 0.107 | 0.136 | 0.118 | 0.115 | 0.115 | 0.117 |
| **VE2_B(m)** | 0.138 | 0.195 | 0.170 | 0.172 | 0.153 | 0.190 |
| **MATS5m** | 0.000 | 0.006 | 0.003 | 0.004 | 0.003 | 0.004 |
| **MATS4v** | 0.001 | 0.003 | 0.002 | 0.002 | 0.001 | 0.003 |
| **MATS5v** | 0.001 | 0.008 | 0.003 | 0.002 | 0.001 | 0.003 |
| **Eig15_EA(dm)** | 0.000 | 0.000 | 0.000 | 0.000 | 0.000 | 0.000 |
| **SPAN** | 8.800 | 10.667 | 9.858 | 10.087 | 8.945 | 10.559 |
| **DISPm** | 13.372 | 18.961 | 15.241 | 14.887 | 13.492 | 15.850 |
| **DISPv** | 5.442 | 7.588 | 6.923 | 7.112 | 6.773 | 7.510 |
| **TDB10m** | 0.090 | 0.115 | 0.106 | 0.109 | 0.101 | 0.114 |
| **TDB10v** | 0.128 | 0.163 | 0.151 | 0.156 | 0.140 | 0.162 |
| **RDF080m** | 0.014 | 1.040 | 0.498 | 0.543 | 0.015 | 0.832 |
| **RDF090m** | 1.021 | 3.291 | 2.285 | 2.392 | 1.647 | 2.970 |
| **RDF100m** | 2.979 | 7.255 | 5.352 | 5.848 | 3.014 | 7.166 |
| **RDF115m** | 0.740 | 2.490 | 1.476 | 1.451 | 0.934 | 1.788 |
| **Mor12u** | -2.755 | -1.421 | -2.075 | -2.139 | -2.344 | -1.653 |
| **Mor22u** | 0.885 | 1.131 | 1.021 | 1.023 | 0.931 | 1.131 |
| **Mor23u** | -2.589 | -2.060 | -2.342 | -2.328 | -2.580 | -2.170 |
| **Mor26u** | 0.359 | 0.530 | 0.467 | 0.478 | 0.440 | 0.514 |
| **Mor32u** | -0.537 | 0.283 | -0.123 | -0.057 | -0.486 | 0.116 |
| **Mor10m** | 0.342 | 0.749 | 0.535 | 0.515 | 0.489 | 0.602 |
| **Mor15m** | -0.002 | 0.173 | 0.088 | 0.086 | 0.048 | 0.137 |
| **Mor24m** | 0.070 | 0.258 | 0.157 | 0.149 | 0.142 | 0.177 |
| **Mor29m** | -0.219 | -0.081 | -0.139 | -0.119 | -0.202 | -0.093 |
| **Mor04v** | -0.135 | 1.475 | 0.490 | 0.416 | 0.224 | 0.546 |
| **Mor27v** | 0.256 | 0.557 | 0.480 | 0.533 | 0.450 | 0.554 |
| **Mor30v** | -0.015 | 0.086 | 0.033 | 0.023 | -0.001 | 0.083 |
| **Mor08p** | 0.014 | 0.176 | 0.113 | 0.132 | 0.076 | 0.147 |
| **Mor11s** | 0.312 | 1.379 | 0.818 | 0.758 | 0.562 | 1.139 |
| **Mor22s** | 2.261 | 2.773 | 2.558 | 2.566 | 2.448 | 2.735 |
| **Mor25s** | 0.673 | 2.052 | 1.169 | 1.101 | 0.854 | 1.234 |
| **Mor28s** | -1.335 | -0.925 | -1.140 | -1.128 | -1.321 | -1.002 |
| **Mor32s** | -1.578 | -0.438 | -0.861 | -0.811 | -0.844 | -0.684 |
| **G1m** | 0.147 | 0.160 | 0.153 | 0.153 | 0.148 | 0.159 |
| **G2m** | 0.169 | 0.211 | 0.184 | 0.181 | 0.174 | 0.188 |
| **E3m** | 0.022 | 0.159 | 0.075 | 0.073 | 0.023 | 0.104 |
| **G2v** | 0.157 | 0.200 | 0.180 | 0.187 | 0.163 | 0.188 |
| **G2e** | 0.156 | 0.199 | 0.182 | 0.182 | 0.174 | 0.198 |
| **E3e** | 0.362 | 0.641 | 0.480 | 0.474 | 0.382 | 0.549 |
| **G2p** | 0.167 | 0.310 | 0.203 | 0.182 | 0.174 | 0.200 |
| **G2s** | 0.148 | 0.245 | 0.196 | 0.203 | 0.155 | 0.220 |
| **Dv** | 0.305 | 0.380 | 0.337 | 0.332 | 0.317 | 0.357 |
| **Dp** | 0.320 | 0.387 | 0.360 | 0.372 | 0.335 | 0.376 |
| **Ds** | 0.472 | 0.596 | 0.545 | 0.548 | 0.512 | 0.593 |
| **H4u** | 2.153 | 3.326 | 2.717 | 2.646 | 2.506 | 3.023 |
| **HATS0v** | 0.038 | 0.055 | 0.047 | 0.046 | 0.044 | 0.052 |
| **HATS0p** | 0.049 | 0.063 | 0.056 | 0.056 | 0.055 | 0.060 |
| **HATS5p** | 0.074 | 0.096 | 0.084 | 0.084 | 0.079 | 0.086 |
| **R1u+** | 0.079 | 0.123 | 0.103 | 0.102 | 0.093 | 0.119 |
| **R3u+** | 0.046 | 0.061 | 0.052 | 0.051 | 0.048 | 0.055 |
| **R4u+** | 0.041 | 0.047 | 0.045 | 0.046 | 0.042 | 0.046 |
| **R5u+** | 0.022 | 0.030 | 0.026 | 0.027 | 0.024 | 0.028 |
| **R4m+** | 0.014 | 0.017 | 0.015 | 0.015 | 0.014 | 0.015 |
| **R1v+** | 0.045 | 0.052 | 0.049 | 0.048 | 0.047 | 0.052 |
| **R1e+** | 0.099 | 0.122 | 0.111 | 0.114 | 0.100 | 0.116 |
| **R3e+** | 0.043 | 0.063 | 0.052 | 0.053 | 0.049 | 0.054 |
| **R7e+** | 0.017 | 0.022 | 0.019 | 0.019 | 0.018 | 0.019 |
| **R1i+** | 0.116 | 0.148 | 0.136 | 0.140 | 0.127 | 0.144 |

**Table S3.** The results of 10-fold CV and external validation

|  | 10-fold CV | External validation |
| --- | --- | --- |
| RMSE | 0.051 |  |
| Q^2^ | 0.987 |  |
| RMSEP |  | 0.050 |
| R^2^ |  | 0.990 |


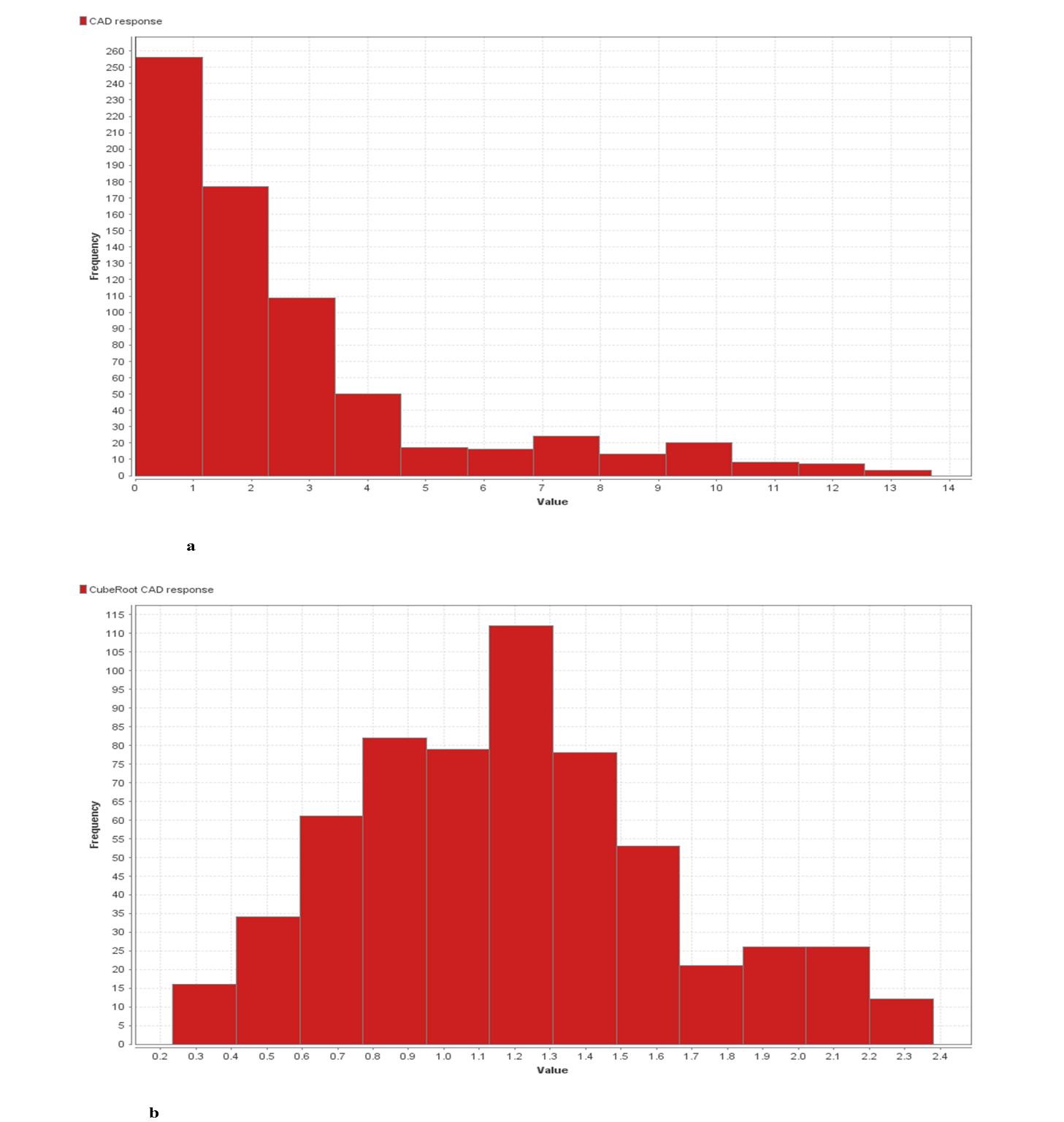


**Fig. S1 Distribution of dependent variable**

1. before cube-root transformation
2. after cube-root transformation


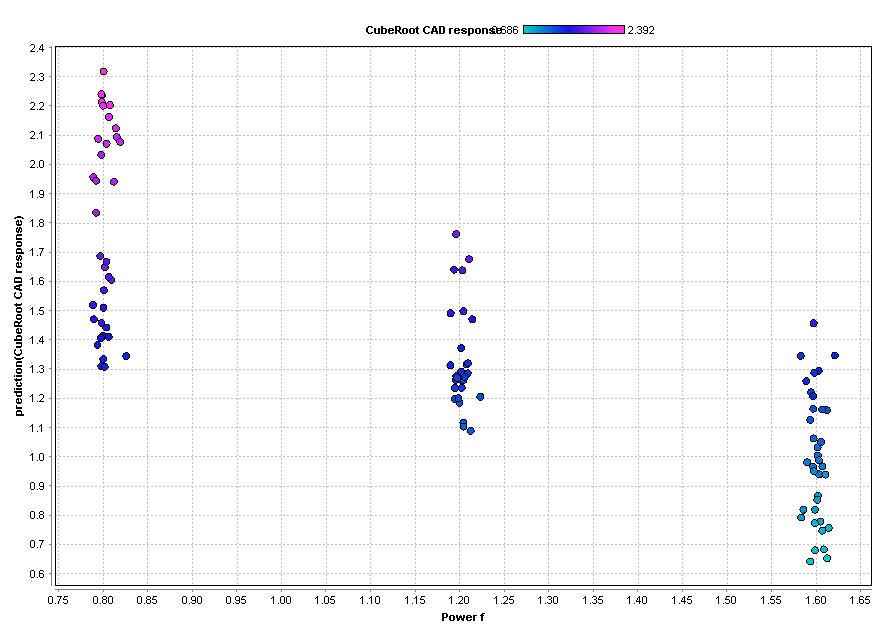


**Fig. S2 Graph showing the relationships between the predicted CAD response and the PFV**


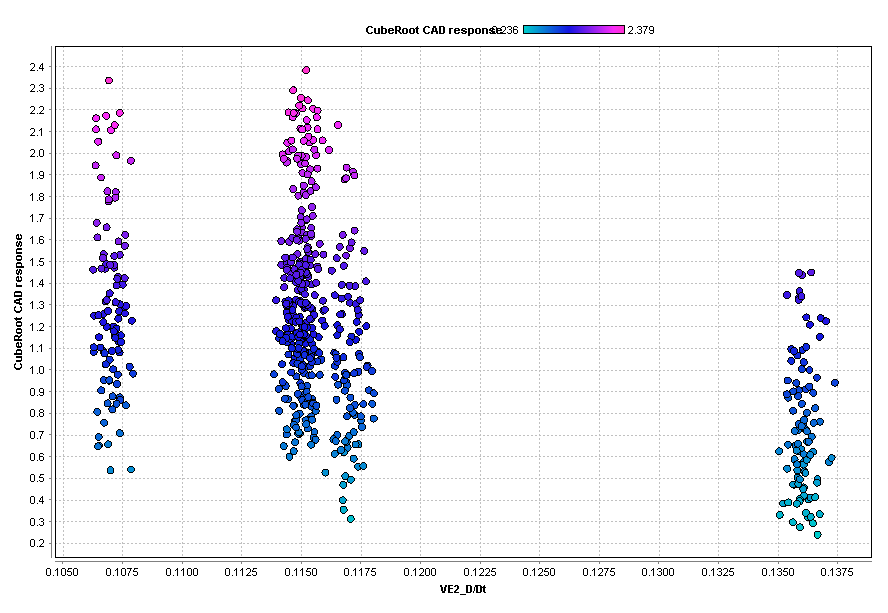


**Fig. S3 Graph showing the relationships between the CAD response of fatty acids and the values of Ve2_D/Dt molecular descriptor**
